# Predicted Health Literacy Disparities Between Immigrant and US-Born Racial/Ethnic Minorities: a Nationwide Study

**DOI:** 10.1007/s11606-023-08082-x

**Published:** 2023-02-27

**Authors:** Aryana Sepassi, Samantha Garcia, Sora Tanjasiri, Sunmin Lee, Mark Bounthavong

**Affiliations:** 1 Department of Clinical Pharmacy Practice, School of Pharmacy & Pharmaceutical Sciences, University of California, Irvine, 802 W Peltason Dr., Irvine, CA 92617 USA; 2 Department of Population and Public Health Sciences, University of Southern California, Los Angeles, CA USA; 3 Keck School of Medicine, Los Angeles, CA USA; 4 Department of Epidemiology & Biostatistics, Program of Public Health, University of California, Irvine, Irvine, CA USA; 5 Department of Medicine, School of Medicine, University of California, Irvine, Irvine, CA USA; 6 Division of Clinical Pharmacy, Skaggs School of Pharmacy & Pharmaceutical Sciences, University of California, San Diego, La Jolla, CA USA; 7 Department of Veteran Affairs, Health Economic Resource Center, Menlo Park, CA USA

**Keywords:** health literacy, racial/ethnic minority, immigrant, health policy

## Abstract

**Background:**

Racial/ethnic minorities in the USA exhibit reduced health literacy (HL) proficiency, leading to increased health disparities. It is unclear how the effect of birth status (immigrant/US-born) affects HL proficiency among racial/ethnic minorities.

**Objective:**

To identify the direct, indirect, and total effects of birth status on HL proficiency among a nationally representative population of racial/ethnic minority adults in the USA.

**Design:**

A cross-sectional study of 2019 data from the Medial Expenditure Panel Survey.

**Participants:**

Participants aged 18 or older reporting as racial/ethnic minorities (Black, Asian, or Hispanic) with non-missing data.

**Main Measures:**

We predicted HL proficiency for each participant using a previously published model. Path analysis was used to estimate the direct, indirect, and total effects of birth status on HL proficiency, accounting for several other covariates. Prevalence ratios were estimated using adjusted Poisson regression to evaluate differences in the “Below Basic” HL category.

**Key Results:**

An estimated weighted 81,092,505 participants were included (57.5% US-born, 42.5% immigrant). More racial/ethnic minority immigrant participants fell into the lowest category of HL proficiency, “Below Basic” (14.3% vs 5.5%, *p* < 0.05). Results of the path analysis indicated a significant, negative direct effect of birth status on HL proficiency (standardized coefficient = − 0.24, SE = 0.01, 95%CI: − 0.26, − 0.23) in addition to an indirect effect mediated through insurance status, health-system resource use, and English proficiency. The total effect of birth status on HL proficiency was found to be − 0.29. The immigrant participant group had 81% higher prevalence of falling into the “Below Basic” HL category compared to US-born participants (prevalence ratio = 1.81, 95%CI: 1.52, 2.16).

**Conclusions:**

Immigrant status has a strong, negative, direct effect on HL proficiency among racial/ethnic minorities in the USA. This may be a result of barriers that prevent equitable access to resources that improve proper HL proficiency. US policymakers may consider several methods to reduce this disparity at the health-system-, provider-, and patient-levels.

**Supplementary Information:**

The online version contains supplementary material available at 10.1007/s11606-023-08082-x.

## BACKGROUND

Approximately 88% of adults in the USA possess limited health literacy (HL) proficiency, leading to difficulty in using health services to maintain quality health behaviors.^1^ The Healthy People 2030 initiative of the Department of Health and Human Services partially defines HL as “the degree to which individuals have the ability to find, understand, and use information and services to inform health-related decisions and actions for themselves and others.”^2^ HL serves as a social determinant of health, mediating the complex factors that play a role in self-management of health behaviors, particularly in chronic disease. Limited HL proficiency has been associated with negative clinical outcomes and reduced disease knowledge in patients with conditions such as congestive heart failure, type II diabetes, asthma, systemic lupus erythematous, and HIV.^3–9^ Moreover, patients with limited HL proficiency are more likely to be admitted for extended inpatient stays, experience avoidable readmissions, and undergo unnecessary emergency care.^10–12^

Racial/ethnic minority populations in the USA exhibit disproportionate rates of limited HL proficiency, with Hispanic or Latino groups demonstrating the lowest rates of basic proficiency or higher, followed by Black or African Americans, and lastly, Asian Americans and Pacific Islanders in aggregate.^1,13,14^ While multiple reasons for these trends may exist, recent research has focused on discriminatory practices and policies that have facilitated the implementation of systematic barriers that prevent these populations from accessing the resources and skills needed to understand and utilize health information in an efficient manner.^15–17^ Included among these barriers are limited educational opportunities, a lack of cultural perspective on health information, mistrust of the health system, and racism.^1,16,18,19^ These barriers have undeniably and unduly facilitated risk of lower HL proficiency in these groups, which has been associated with a lack of insurance coverage, overestimation of HL proficiency by physicians, and reduced use of preventive care.^20–22^

English proficiency is a crucial factor associated with HL proficiency that is often underscored in studies of racial/ethnic minority groups. Among racial/ethnic minorities with low HL proficiency, 44.9% report limited English proficiency, versus 13.8% of those who speak fluent English.^22^ Racial/ethnic minority immigrants exhibit this trend more so than their US-born equivalents, with 40% demonstrating limited English proficiency, and 60% reporting bilingual use at home.^23^ In contrast, only 8% of the total US population reports limited English proficiency, and 21.9% speak a language other than English at home.^24,25^ Racial/ethnic minority immigrants may therefore be at an especially higher risk of demonstrating lower HL proficiency than their US-born counterparts. Limited English proficiency in racial/ethnic minority immigrants may compound the vulnerabilities discussed above, such as educational attainment, underuse or misuse of the healthcare system, and reduction in health literacy proficiency. However, the magnitude of this association has not been previously quantified. Therefore, an analysis of the differences in HL proficiency between immigrant and US-born racial/ethnic minority groups is warranted to better understand predictors of low HL and to inform future interventions and eliminate barriers to obtaining adequate HL proficiency in this population. The primary aim of this study was to analyze the magnitude of association between birth status and HL proficiency between US-born and immigrant racial/ethnic minority groups at the national level. Our secondary aim was to estimate differences in prevalence between the US-born and immigrant racial/ethnic minorities for a “Below Basic” HL category.

## METHODS

### Data Source and Study Population

This cross-sectional study was approved by the Institutional Review Board (IRB) at the University of California, Irvine and involved a retrospective review of existing de-identified data. We used data from the Full-Year Consolidated (FYC) 2019 Medical Expenditure Panel Survey (MEPS) file, from the federal Agency for Healthcare Research and Quality (AHRQ). MEPS is a nationally representative survey of the US civilian, noninstitutionalized population that collects a variety of demographic and health-related survey data on demographic characteristics, health conditions/diagnoses, use of medical care services, and other related data.^28,29^ Complex survey design methods are used to yield weighted estimates using clustering, stratification, and multistage and disproportional sampling, with oversampling of minorities to allow for nationally representative estimates.^29^ The MEPS surveys are administered in English and Spanish, with other languages available at the participant’s request. Each interview contains a series of computer-assisted personal interview screens with questions, instructions, and skip patterns based on specific topics. We limited our analysis to racial/ethnic minority participants in rounds of the 2019 MEPS data self-reporting their race or ethnicity as exclusively “Hispanic or Latino,” “Non-Hispanic Black or African American,” or “Non-Hispanic Asian American or Pacific Islander” and aged 18 years or older. Immigrants were identified as participants responding “No” to the question “Were you born in the United States?”.

### Measures: Estimated Health Literacy Proficiency

We estimated each participant’s HL score using a previously published linear regression model.^30^ The model was developed and validated using data from the 2003 National Assessment of Adult Literacy (NAAL) study, which evaluated written HL proficiency.^1,30^ Each participant’s HL score was predicted using a series of demographic variables including gender, age, race/ethnicity, education, income, marital status, language spoken at home, rurality, and time in the USA.^30^ The published model accounted for 30% of the variance in NAAL-derived HL scores.^30^ We evaluated HL scores as continuous and categorical variables. Categories were defined using the 2003 NAAL criteria, where a score of 0–184 is “below basic,” 185–225 is “basic,” 226–309 is “intermediate,” and 310–500 is “proficient.”.^1^

### Measures: Demographic Variables

We assessed demographic differences among US-born and immigrant groups. Age was categorically described in years (18–24/25–39/40–49/50–64/65–74/75 +), metropolitan statistical area (MSA) status was stratified by MSA/non-MSA, and sex was categorized as male/female. Geographic location was categorized as Northeast, Midwest, South, and West. Marital status was defined as married, widowed, divorced, separated, or never married. Primary language spoken at home was categorized as English, Spanish, or other. Among those not reporting English as a primary language, we included self-reported English proficiency as speaking very well, well, not well, or not at all. Self-reported physical status was included as excellent, very good, good, fair, or poor. Years in the USA were categorized as born in the USA, less than 5 years, 6 to 10 years, and 10 years or more. Cognitive limitations, visual difficulties, and hearing difficulties were categorized as yes/no responses (Supplemental Materials). Employment status was categorized as employed/not employed. Income level was categorized as poor/negative, near poor, low income, middle income, and high income. Insurance coverage status was categorized as any private, public only, or uninsured. Education was reported as the highest degree obtained when entering MEPS (don’t know, no degree, GED, HS diploma, bachelor’s degree, master’s degree, doctorate, other degree). Charlson comorbidity index was calculated using a previously published algorithm using ICD-10 codes from the 2019 Medical Conditions supplemental file to account for disease burden.^31^

### Statistical Analysis

**Fig. 1:**
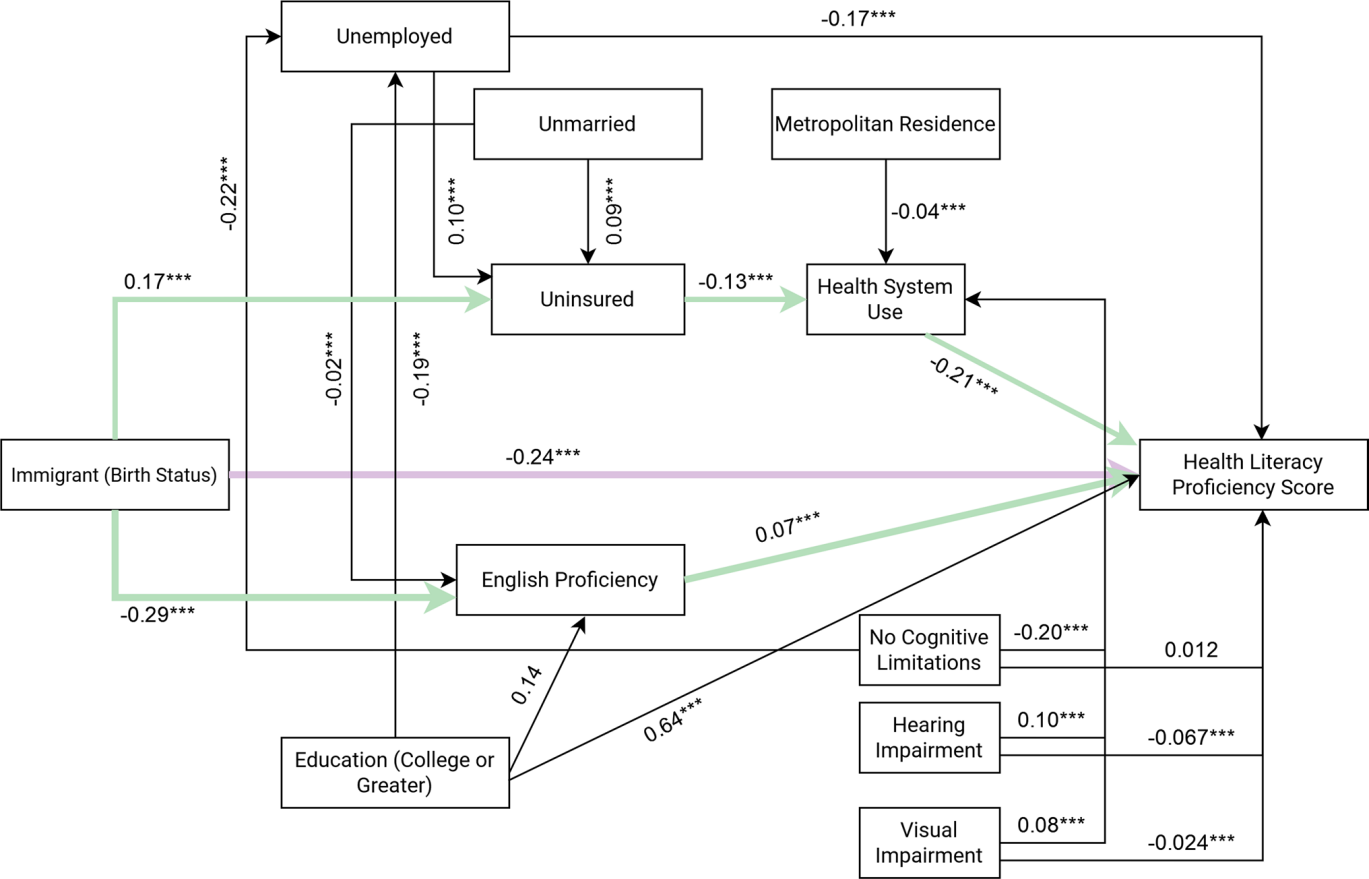
Path analysis model. Theoretical path analysis model evaluating interrelated variables and their direct and indirect effects on health literacy proficiency. Direct effect standardized coefficients are explicitly stated. The direct effect pathway from immigrant birth status to predicted health literacy proficiency score is denoted by the lilac arrow. Indirect pathways are denoted by green arrows. ***: denotes *p* < 0.05

Descriptive statistics are presented as means and standard deviations (SD) for continuous data, and frequencies with proportions for categorical data. Chi-square tests for categorical data and Kruskal–Wallis tests for continuous data were used to report differences between the US-born and immigrant groups. Patient-level weights were applied to account for complex survey design and generate estimates for the representative US population. Participants with missing data (*n* = 61 unweighted, 1.2%) were excluded from analysis.^32^ Variance inflation factor (VIF) and tolerance values were computed to verify no signs of potential multicollinearity between variables. A *p*-value of < 0.05 was considered statistically significant. All analyses were performed using STATA (College Station, TX). To test for the direct, indirect, and total effects of birth status on health literacy score, we developed a path analysis model using the maximum likelihood method and STATA’s *sem* command (Fig. 1). Healthcare burden and use was measured using the following observed variables: number of outpatient visits, number of emergency room (ER) visits, total health expenditure, self-reported physical health, number of drugs purchased, age, number of office-based visits, and number of inpatient visits. To ensure healthcare burden and use measures were appropriate, a latent measurement model was first constructed to test psychometric properties. After establishing the model, healthcare burden and use was introduced as an observed variable. In the path analysis, a parsimonious model was constructed by eliminating non-significant model paths. Satorra-Bentler adjustments were used for model output to account for non-normality of HL score distribution and to estimate robust standard error. Model fit was assessed based on multiple indicators: Comparative Fit Index (CFI), root mean square error approximation (RMSEA), Tucker-Lewis coefficient (TLI), root mean square residual (SRMR), and Akaike’s Information Criterion (AIC, for determining best fit among multiple model iterations).^33–35^ CFI and TLI values of 0.95 or greater and RMSEA and SRMR values of 0.08 or less were determined to be good model fits.^33–35^ Direct effects between variables were measured as standardized coefficients in pathways between those variables. Indirect effects between variables were measured along pathways between variables that may contain one or more mediators (Fig. 1). Our secondary aim was assessed using a Poisson regression model that was fitted to estimate the prevalence ratio (PR) of falling into a “Below Basic” HL score category among immigrant vs US-born participants.^36^ The model was adjusted for covariates that included age, sex, race/ethnicity, marital status, income, English proficiency, insurance status, comorbidities, education, and employment.

## RESULTS

Our final analytic sample consisted of 8,486 unweighted participants, corresponding to weighted 81,092,505 participants. US-born participants constituted 57.5% of the cohort. Demographic differences between immigrant and US-born racial/ethnic minorities are described in Table 1. Both immigrant and US-born groups followed similar frequency distributions for metropolitan status, geographic location, sex, self-reported physical status, cognitive limitations, difficulty hearing, difficulty seeing, employment status, income category, and insurance coverage. More immigrant participants were Asian or Pacific Islander (33.3% vs 6.3% immigrant vs US-born) or Hispanic (57.3% vs 40.4% immigrant vs US-born). Conversely, non-Hispanic Black or African American participants tended to report as US-born (53.4% vs 9.4% US-born vs immigrant). More immigrant participants reported as being married (63.4% vs 33.6% immigrant vs US-born). Fewer immigrant participants reported speaking English as a primary language (11.4% vs 72.2% immigrant vs US-born). Of these, 35.1% reported speaking English as “Very Well.” More immigrants reported as having been in the USA for at least 10 years (80.3%) in addition to reporting themselves as uninsured (18.0% vs 10.2% immigrant vs US-born). The majority of both groups reported as having a high school diploma at the time of entering MEPS, but more immigrants reported themselves as having no degree (27.9% vs 15.6% immigrant vs US-born). Mean predicted HL score for the US-born group was significantly higher than the immigrant group (226.6 (0.58) vs 213.5 (0.99) mean (SD), *p* < 0.05, respectively). Differences in predicted HL scores between the two groups according to NAAL definitions of categories are presented in Table 2. More immigrant participants fell into the “Below Basic” category than US-born respondents (14.3% vs 5.5%, *p* < 0.05).

**Table 1:** Survey Weighted Baseline Demographics Among US-Born and Immigrant Racial/Ethnic Minorities

	US-born (*N* = 46,646,844)	Immigrant (*N* = 34,445,661)	*p*-value
	*N* (%)	*N* (%)	
Age (years)			
18–24	7,379,483 (15.8)	1,750,308 (5.1)	< 0.05
25–39	16,621,854 (35.6)	10,179,416 (29.6)	
40–49	7,034,592 (15.1)	8,333,079 (24.2)	
50–64	9,084,953 (19.5)	9,057,147 (26.2)	
65–74	4,225,705 (9.1)	3,073,448 (8.9)	
75 +	2,300,258 (4.9)	2,052,264 (6.0)	
Metropolitan status			
Non-MSA^*^	3,430,243 (7.3)	1,037,491 (3.0)	< 0.05
Geographic location			
Northeast	5,912,779 (16.7)	6,605,510 (19.2)	< 0.05
Midwest	6,463,063 (13.9)	3,696,331 (10.7)	
South	22,218,785 (47.6)	12,760,702 (37.1)	
West	12,052,217 (25.8)	11,383,118 (33.1)	
Sex			
Male	22,011,609 (47.2)	16,618,044 (48.2)	0.30
Race/ethnicity			
Hispanic or Latino	18,831,838 (40.4)	19,736,474 (57.3)	< 0.05
Non-Hispanic Black or African American	24,896,278 (53.4)	3,248,927 (9.4)	
Non-Hispanic Asian American or Pacific Islander	2,918,728 (6.3)	11,460,261 (33.3)	
Marital status			
Married	15,689,644 (33.6)	21,836,230 (63.4)	< 0.05
Widowed	2,179,837 (4.7)	1,653,306 (4.8)	
Divorced	5,295,635 (11.4)	2,371,895 (6.9)	
Separated	1,317,722 (2.8)	1,047,723 (3.0)	
Never married	2,2164,006 (47.5)	7,536,507 (21.9)	
Primary language spoken at home			
English	33,692,082 (72.2)	3,910,622 (11.4)	< 0.05
Spanish	11,435,027 (24.5)	18,473,525 (53.6)	
Other	1,519,735 (3.3)	1,2061,515 (35.0)	
English proficiency			
Inapplicable (speaks English at home)	33,692,082 (72.2)	3,910,622 (11.4)	< 0.05
Very well	11,296,714 (24.2)	12,080,130 (35.1)	
Well	1,208,288 (2.6)	7,341,035 (21.2)	
Not well	361,555 (0.8)	7,510,290 (21.8)	
Not at all	88,206 (0.2)	3,603,585 (10.5)	
Self-reported physical health			
Excellent	12,367,013 (26.5)	9,098,026 (26.4)	< 0.05
Very good	13,937,646 (29.9)	9,856,778 (28.6)	
Good	13,769,789 (29.5)	10,645,382 (30.9)	
Fair	5,326,406 (11.4)	4,130,335 (12.0)	
Poor	1,245,990 (2.7)	715,141 (2.1)	
Years in the USA			
Born in the USA	4,6646,844 (100.0)	–	< 0.05
< 5 years	–	2,939,468 (8.5)	
6–10 years	–	3,836,723 (11.2)	
Over 10 years	–	27,669,470 (80.3)	
Cognitive limitations			
Don’t know	180,264 (0.4)	99,494 (0.3)	< 0.05
Inapplicable	1,360,661 (2.9)	170,546 (0.5)	
Yes	2,498,641 (5.4)	1,177,481 (3.4)	
No	42,607,277 (91.3)	32,998,140 (95.8)	
Difficulty hearing			
Yes	1,292,009 (2.8)	758,488 (2.2)	< 0.05
Difficulty seeing			
Yes	1,409,554 (3.0)	512,213 (1.5)	< 0.05
Employment status			
Employed	29,783,524 (63.6)	23,371,505 (67.8)	< 0.05
Income category^†^			
Poor/negative	7,518,325 (16.1)	4,702,921 (13.6)	0.25
Near poor	2,317,113 (5.0)	1,706,061 (5.0)	
Low income	7,223,617 (15.5)	5,571,585 (16.2)	
Middle income	14,900,526 (31.9)	10,960,846 (31.8)	
High income	14,687,263 (31.5)	6,185,514 (33.4)	
Insurance coverage			
Any private	28,523,642 (61.2)	19,427,453 (56.4)	< 0.05
Public only	13,388,299 (28.7)	8,832,695 (25.6)	
Uninsured	4,732,903 (10.1)	6,185,514 (18.0)	
Highest degree earned			
Don’t know	71,917 (0.2)	28,849 (0.1)	< 0.05
No degree	7,265,200 (15.6)	9,608,396 (27.9)	
GED^‡^	2,109,248 (4.5)	763,052 (2.2)	
High school diploma	21,513,826 (46.1)	10,686,363 (31.0)	
Bachelor’s degree	7,306,580 (15.7)	6,581,581 (19.1)	
Master’s degree	3,077,054 (6.6)	3,742,232 (10.9)	
Doctorate degree	541,654 (1.2)	845,863 (2.5)	
Other degree	4,761,365 (10.2)	2,189,365 (6.4)	
Charlson comorbidity index score (mean, SD)	1.9 (25.9)	1.7 (29.5)	0.05

^*^MSA metropolitan statistical area

^†^Income category definitions: poor/negative: income ≤ poverty line, near poor: income > poverty line through 125%, low income: income > 125% through 200%, middle income: income > 200% through 400%, high income: income > 400%

^‡^*GED* General Education Development

**Table 2:** Predicted Health Literacy Scores, Racial/Ethnic Minority US-Born Participants vs Immigrant Participants

Health literacy category	US-born (*N* = 46,646,844)	Immigrant (*N* = 34,445,661)
	*N* (%)	*N* (%)
Below basic	2,585,538 (5.5)	4,913,761 (14.3)
Basic	19,857,303 (42.6)	16,718,545 (48.5)
Intermediate or greater	24,204,003 (51.9)	12,813,355 (37.2)

Below basic corresponds to predicted scores between 0 and 184. Basic corresponds to predicted scores between 185 and 225. Intermediate or greater corresponds to predicted scores between 226 and 500

Our fit of the latent variable “healthcare burden and use” using Confirmatory Factor Analysis (CFA) was acceptable. Internal consistency was adequate (Cronbach’s alpha = 0.743) for all factors. Additional fit indices indicated an acceptable fit (RMSEA = 0.064, CFI = 0.951, TLI = 0.932). The *R*^2^ value for the latent variable was 0.85, indicating the fit explained 85% of the variance in the fitted observed variables. The data fit adequately for the final path analysis model (RMSEA = 0.059, SRMR = 0.031, CFI = 0.957, TLI = 0.903), indicating that the data used in our study were consistent with our theoretical model. The chi-square test of model fit was significant (*p* < 0.001). However, this significance was likely due to the large number of parameters estimated and large sample size.^33^ Table 3 displays standardized direct path coefficients. Analyses on direct effects indicated that immigrant birth status had a significant, negative direct effect on predicted HL score [standardized coefficient = − 0.24, SE = 0.006, 95%CI: − 0.26, − 0.23, *p* < 0.05]. Moreover, immigrant birth status maintained significant negative indirect effects that were mediated through insurance status, health system resource use and burden, and English proficiency [standardized coefficient = − 0.18, SE = 0.13, *p* < 0.05]. Consequently, the total effect of immigrant birth status on predicted HL score was − 0.26, indicating a negative relationship between immigrant birth status and predicted HL score. Notably, education (high school or greater) was the only other variable with a direct effect of stronger magnitude on predicted HL score than birth status [standardized coefficient = 0.64, SE = 0.01, 95%CI: 0.63, 0.65, *p* < 0.05]. Our model explained up to 67.2% of the variance in HL score.

**Table 3:** Results of Path Analysis: Standardized Direct Effect Path Coefficients

Parameter	Standardized coefficient (standard error; SE)	95% confidence interval	
		Lower bound	Upper bound
To English proficiency			
Immigrant birth status	− 0.29*** (0.01)	− 0.31	− 0.27
Education (HS or more)	0.14*** (0.00)	0.12	0.16
Unmarried	− 0.02*** (0.01)	− 0.043	− 0.00084
To predicted HL score			
English proficiency (very well or higher)	0.07*** (0.07)	0.067	0.088
Unemployed	− 0.17*** (0.0069)	− 0.19	− 0.16
Health system resource use and burden	− 0.21*** (0.0089)	− 0.22	− 0.19
Immigrant birth status	− 0.24*** (0.0064)	− 0.26	− 0.23
Education (HS or more)	0.64*** (0.0051)	0.63	0.65
No cognitive limitations	0.012 (0.0067)	− 0.00027	0.026
Visual limitations	− 0.024*** (0.0064)	− 0.037	− 0.012
Hearing limitations	− 0.067*** (0.0065)	− 0.080	− 0.055
To unemployment			
Education (HS or more)	− 0.19*** (0.01)	− 0.21	− 0.17
No cognitive limitations	− 0.22*** (0.01)	− 0.24	− 0.20
To insurance coverage			
Unemployed	0.10*** (0.04)	0.02	0.19
Immigrant birth status	0.17*** (0.01)	0.15	0.19
Unmarried	0.09*** (0.01)	0.07	0.11
To health system resource use and burden			
Insurance coverage	− 0.13*** (0.01)	− 0.16	− 0.11
No cognitive limitations	− 0.20*** (0.01)	− 0.22	− 0.18
Visual limitations	0.08*** (0.01)	0.06	0.11
Hearing limitations	0.10*** (0.01)	0.08	0.12
Metropolitan residence	− 0.04*** (0.01)	− 0.06	− 0.02

^***^p < 0.05; *HS* high school; *HL *health literacy

The results of the adjusted Poisson regression model can be found in Table 4. Immigrants were estimated to have 1.81 times the prevalence of participants falling into a “Below Basic” HL category compared to US-born racial-ethnic minority participants [PR = 1.81, SE = 0.16, 95%CI (1.52, 2.16), *p* < 0.05]. Notably, females had a lower prevalence ratio compared to males [PR = 0.63, SE = 0.05, 95%CI (0.55, 0.73), *p* < 0.05]. Compared to Hispanic or Latinos, Black or African American participants reported a higher prevalence ratio, with Asian American or Pacific Islanders reporting a lower ratio. Increasing income category also reduced prevalence ratio estimates compared to those in the poorest category. Unemployed participants and participants without insurance coverage reported higher prevalence rates than employed and insured participants, respectively. Prevalence decreased significantly as level of education increased.

**Table 4:** Poisson Regression Results for Deriving Prevalence Ratio Between Immigrant and US-Born Participants on Falling into “Below Basic” Predicted Health Literacy Category

Parameter	Coefficient (standard error; SE)	95% confidence interval	
		Lower bound	Upper bound
Birth status			
US-born	Reference		
Immigrant	1.81*** (0.16)	1.52	2.16
Age (continuous)	1.08*** (0.004)	1.07	1.09
Sex			
Male	Reference		
Female	0.63*** (0.05)	0.55	0.73
Race/ethnicity			
Hispanic or Latino	Reference		
Black or African American	1.38*** (0.12)	1.17	1.64
Asian American or Pacific Islander	0.51*** (0.08)	0.39	0.68
Marital status			
Married	Reference		
Never married	1.05 (0.11)	0.85	1.29
Divorced, separated, or widowed	1.05 (0.08)	0.90	1.22
Income category			
< 100% FPL	Reference		
100–200% FPL	0.79*** (0.05)	0.70	0.89
200–300% FPL	0.77*** (0.07)	0.64	0.92
≥ 300% FPL	0.41*** (0.05)	0.33	0.51
English proficiency			
Very well	Reference		
Well	1.11 (0.17)	0.82	1.51
Not well	1.15 (0.15)	0.89	1.50
Not at all	1.20 (0.17)	0.91	1.57
Insurance coverage			
Private only	Reference		
Public only	1.21 (0.12)	0.99	1.48
Uninsured	1.44*** (0.23)	1.05	1.97
CCI score (continuous)	0.95*** (0.02)	0.90	0.99
Education			
Less than high school	Reference		
High school	0.50*** (0.03)	0.44	0.57
College	0.02*** (0.008)	0.006	0.04
Graduate or other	0.02*** (0.02)	0.006	0.09
Employment status			
Employed	Reference		
Unemployed	1.21 (0.13)	0.98	1.49

^***^p < 0.05; *FPL* federal poverty line, *CCI* Charlson comorbidity index

## DISCUSSION

We reported that low HL was influenced by several predictors that include racial/ethnic minority immigrant birth status, low education, low income, and unemployment. The multidimensional nature of HL demonstrated herein mirrors previous research in the USA. For example, Martin et al. (2009) analyzed data from the 2003 National Assessment of Adult Literacy (NAAL) and reported significant predictors of low HL included gender, age, race/ethnicity, educational attainment, poverty, and language spoken at home.^30^ Houston et al. (2019) reported that higher education and Latino cultural identification predicted English HL, with education also accounting for the relationship between income and HL; Sentell and Braun (2012) reported greater proportions of low HL among limited English proficient Chinese (68.3%), Latinos (45.3%), Koreans (35.6%), and Vietnamese (29.7%) compared to Whites (18.8%).^37,38^

Our results suggest a complex interrelationship between HL proficiency, birth status, socioeconomic status, education, and employment. Furthermore, we found that racial/ethnic minority immigrants had a higher prevalence of being categorized with “Below Basic” proficiency. These results may be indicative of underlying inequities faced by racial/ethnic minority immigrants, which in turn reduce opportunities for achieving crucial factors for development of higher HL proficiency, such as social and economic upward mobility. For example, 14% of Black immigrants live below the poverty line, higher than the average US population (11%).^39^ In general, 20% of immigrants are unemployed in the US, also higher than the average US population rate of 13%.^39^ Asian and Hispanic immigrant groups are also more likely to have less than a high school education compared to their US-born counterparts, at 10% vs 3% and 55% vs 21% immigrant vs US-born, respectively.^40^ We argue that the inequities in these social determinants of health are upstream influencers of health literacy proficiency, as our findings suggest. We encourage future investigators to assess the effects of these determinants of health on HL proficiency more closely among racial-ethnic minority immigrants to better understand their relationships.

Our study findings carry potential implications from a US health policy perspective for racial/ethnic minority immigrants struggling with low HL proficiency. Contrary to US-born racial/ethnic minorities, racial/ethnic minority immigrants are highly heterogeneous with respect to socioeconomic status, English proficiency, and other factors that affect their reduced access to care and risk of negative health outcomes. This is further compounded by policies such as the 1996 Personal Responsibility Work Opportunity Reconciliation Act (PRWORA), which restricted immigrants’ eligibility for federally funded welfare programs, such as Medicaid.^41^ These inequities are also likely to be associated with the low HL proficiency among racial/ethnic minority immigrants that we observed herein. To address this issue and promote downstream improvements in HL, several immediate policy changes may be considered at the health-insurer, organizational, and provider levels.

One may consider improving access to language services at the health-system level. The National Standards for Culturally and Linguistically Appropriate Services (CLAS) in health care, as well as recent initiatives such as the National Action Plan, address this limitation to some extent, with the objective to improve English language instruction and to provide culturally and linguistically appropriate health information services in the community.^42,43^ Other more direct policy changes may include health insurers incentivizing promotion of HL from providers by incorporating goals into payment systems, such as with usage of translation and interpreter services, as is the case with Medicare. This may be especially appropriate for Medicaid/CHIP, which may consider requiring programs to cover access to necessary interpreter services in the 38 states that currently do not cover use of interpreter or translative services.^44^ Expansion of reimbursement mechanisms may also benefit racial/ethnic minority immigrants covered by commercial or private third-party payer plans, which are also exempt from Title VI of the Civil Rights Act of 1964.^44^ This initiative has been supported by the American Medical Association, citing these services as improving patient-provider trust.^45^ Another relatively non-disruptive method may be to integrate health literacy skills as a fundamental component of health professional education and training programs. Research has demonstrated that health science students also lack considerable proficiency in HL themselves, with 30.2% reporting inadequate or problematic HL.^46^ Coleman and colleagues demonstrated that healthcare professionals overestimate their understanding of HL limitations from patients, and with a proper, targeted intervention, these understandings may become more realistic.^47,48^ Several studies have demonstrated the effectiveness in improving awareness of HL disparities in providers after training interventions as promoting knowledge, behaviors, and confidence in using HL strategies with patients and their families.^49–52^

Parallel to these potential changes in health policy to promote language inclusivity for immigrants of racial/ethnic minority backgrounds, there are additional policy changes related to education, welfare, and employment that may benefit to improve health literacy proficiency. For example, the Illegal Immigration Reform and Immigrant Responsibility Act (IIRAIRA) of 1996 implemented irrevocable adverse changes to immigrant welfare in the USA subsequent to the PRWORA, including prevention of states from providing post-secondary education benefits to most groups of “non-citizens,” including non-citizen immigrants.^53^ To date, 21 states and the District of Columbia have enacted policies to reverse this impact, allowing any individual to apply to state-level funding for post-secondary education, regardless of citizenship or immigration status.^54^ Reversal of policies such as this may serve to improve access to the predictors of below basic HL proficiency that we have demonstrated herein, such as education and employment. Moreover, more recent action such as the Deferred Action for Childhood Arrivals (DACA) from 2012, which has demonstrated positive financial and health outcomes for immigrant children, may serve to benefit from implementing a pathway to expedited citizenship after an immigrant student’s 2-year provisional period is over, contingent on fulfillment of educational requirements.^55,56^ In addition, policies that currently exclude immigrants from pursuing higher wage professions may also drive the negative downstream effects on HL proficiency that we have noted in our study, such as requirements for proof of citizenship in order to obtain professional licenses (e.g., optometry, physical therapy, real estate).^57^ Several states have reversed such policies, which may improve access to health insurance coverage in addition to economic self-sufficiency, which in turn may drive improvements in health literacy proficiency.^58^

This study had limitations of note. Our data source, MEPS, is cross-sectional data. Therefore, the directionality of the paths in our path analysis model is only hypothesized. For some cases, one may argue that the reverse paths are also true, such as the case with health system utilization as influencing HL proficiency. While our sample size in total was relatively large, the majority of respondents were Hispanic or Latino. Therefore, our results may be less precise for Asian American or African American populations. The model that we utilized to predict HL score explained 29.8% of the total variance in mean health literacy score.^30^ Thus, true HL scores may have varied from our predictions of the MEPS population. This may have been due to the exclusion of other hypothesized predictors of HL proficiency described in various HL frameworks that were not present in MEPS data, including cultural values or beliefs, ability to access information, strength of social support networks, memory ability and reasoning, reading fluency, and prior knowledge of the healthcare system.^59–61^ The same model also validated prediction of health literacy scores using NAAL data from 2003, which distinctly evaluated HL reading comprehension.^1^ Therefore, the results of our study should only be taken in the context of HL reading comprehension, instead of including verbal comprehension or numeracy. Our study groups also contained significant differences at baseline. For example, the racial/ethnic minority immigrant group on average had a higher proportion of Asian Americans or Pacific Islanders, with the opposite as true for the US-Born group. This may have skewed average HL scores between the two groups. Finally, our results are only generalizable to Asian American/Pacific Islander, Hispanic or Latino, and Black or African American immigrant communities, as we did not account for other groups that may immigrate to the US with higher average levels of education, employment, etc. (e.g., White immigrants).

## CONCLUSION

Among racial/ethnic minorities, birth status has a significantly negative direct and indirect effect on predicted health literacy proficiency, with minority immigrants reporting higher prevalence of “Below Basic” proficiency. These results are reflective of a combination of upstream inequities in social determinants of health and health policy. Policymakers should consider implementation of changes aimed at improving access to more equitable health literate services specifically targeted toward racial/ethnic minority immigrants to fill these gaps.

## Supplementary Information

Below is the link to the electronic supplementary material.

Supplementary file1 (DOCX 57 KB)

## Data Availability

The datasets generated during and/or analyzed during the current study are not publicly available. Due to the use of MSA data in this study, the data are restricted such that only the Agency for Healthcare Research and Quality (AHRQ) have access due to potential concerns on re-identification of participant records.

## References

[1] **Kutner M, Greenberg E, Jin Y, Paulsen C.** The Health Literacy of America’s Adults: Results from the 2003 National Assessment of Adult Literacy.” 2006. US Department of Education, National Center for Education Statistics.

[2] **Health People 2030.** Office of Disease Prevention and Health Promotions. (n.d.). *Healthy People 2030.* US Department of Health and Human Services.

[3] Cajita MI, Cajita TR, Han HR (2016). Health Literacy and Heart Failure: A Systematic Review. J Cardiovasc Nurs..

[4] Caruso R, Magon A, Baroni A (2018). Health literacy in type 2 diabetes patients: a systematic review of systematic reviews. Acta Diabetol..

[5] Gazmararian JA, Williams MV, Peel J (2003). Health literacy and knowledge of chronic disease. Patient Educ Couns..

[6] Kalichman SC, Benotsch E, Suarez T (2000). Health literacy and health-related knowledge among persons living with HIV/AIDS. Am J Prev Med..

[7] Maheswaranathan M, Cantrell S, Eudy AM, Rogers JL, Clowse MEB, Hastings SN (2021). Investigating Health Literacy in Systemic Lupus Erythematosus: a Descriptive Review. Curr Allergy Asthma Rep..

[8] Marciano L, Camerini AL, Schulz PJ (2019). The Role of Health Literacy in Diabetes Knowledge, Self-Care, and Glycemic Control: a Meta-Analysis. J Gen Intern Med..

[9] **Williams MV, Baker DW, Parker RM, et al.** Relationship of functional health literacy to patients’ knowledge of their chronic disease. A study of patients with hypertension and diabetes. *Arch Intern Med*. 1998;158(2):166–72. 10.1001/archinte.158.2.166 9448555

[10] Baker DW, Wolf MS, Feinglass J (2007). Health Literacy and Mortality Among Elderly Persons. Arch Intern Med..

[11] Berkman ND, Sheridan SL, Donahue KE (2011). Low health literacy and health outcomes: an updated systematic review. Ann Intern Med..

[12] Cho YI, Lee SD, Arozullah AM (2008). Effects of health literacy on health status and health service utilization amongst the elderly. Soc Sci Med..

[13] Kreps GL, Sparks L (2008). Meeting the health literacy needs of immigrant populations. Patient Educ Couns..

[14] **Park SY, Lee H, Kang M.** Factors affecting health literacy among immigrants – systematic review. *European Journal of Public Health*. 2018;28(Suppl 4)

[15] Foulk D, Carroll P, Wood SN (2001). Addressing Health Literacy: a description of the intersection of functional literacy and health. American Journal of Health Statistics..

[16] Prins E, Mooney A (2014). Literacy and health disparities. New Directions for Adult and Continuing Education..

[17] Smith AL (2003). Health policy and the coloring of an American male crisis: A perspective on community-based health services. American Journal of Public Health..

[18] Muramastu N, Marshall C (2022). Battling Structural Racism Against Asians in the United States: Call for Public Health to Make the “Invisible” Visible. Journal of Public Health Management and Practice..

[19] Goodman MS, Gaskin DJ, Si X (2012). Self-reported segregation experience throughout the life course and its association with adequate health literacy. Health and Place..

[20] Edward J, Wiggins A, Young MH (2019). Significant Disparities Exist in Consumer Health Insurance Literacy: Implications for Health Care Reform. Health Lit Res Pract..

[21] Kelly PA, Haidet P (2007). Physician overestimation of patient literacy: a potential source of health care disparities. Patient Educ Couns..

[22] Sentell TL, Tsoh JY, Davis T (2015). Low health literacy and cancer screening among Chinese Americans in California: a cross-sectional analysis. BMJ Open..

[23] **US Census Bureau.** 2012. 2010 American Community Survey. Accessed from steven Ruggles, Katie Genadek, Ronald Goeken, Josiah Grover, and Matthew Sobek. Integrated Public Use Microdata Series: Version 6.0 [Machine-readable database]. Minneapolis: University of Minnesota.

[24] **Center for Immigration Studies.** *67.3 million in the United States Spoke a Foreign Language at Home in 2018*. https://cis.org/sites/default/files/2019-10/camarota-language-19_0.pdf. Published 2019. Accessed November 10, 2022.

[25] **Migration Policy Institute.** *The Limited English Proficient Population in the United States in 2013*. https://www.migrationpolicy.org/article/limited-english-proficient-population-united-states-2013. Published 2015. Accessed November 10, 2022.

[26] Toppelberg CO, Collins BA (2010). Language, Culture, and Adaptation in Immigrant Children. Child Adolesc Psychiatr Clin N Am..

[27] **Budiman A, Ruiz NG.** Key facts about Asian Americans, a diverse and growing population. Pew Research Center. April 29, 2021. Accessed October 4, 2022. Key facts about Asian Americans | Pew Research Center

[28] **Agency for Healthcare Research and Quality.** Medical Expenditure Panel Survey. MEPS 2019 Full-Year Consolidated Data File. Available from: https://www.meps.ahrq.gov/mepsweb/data_stats/download_data_files_detail.jsp?cboPufNumber=HC-216. Accessed December 2^nd^, 2021.

[29] **Chowdhury SR, Machin SR, Gwet KL.** Sample Designs of the Medical Expenditure Panel Survey Household Component, 1996–2006 and 2007–2016. Methodology Report #33. January 2019. Agency for Healthcare Research and Quality, Rockville, MD. https://meps.ahrq.gov/data_files/publications/mr33/mr33.shtml

[30] Martin LT, Ruder T, Escarce JJ, Ghosh-Dastidar B, Sherman D, Elliott M (2009). Developing Predictive Models of Health Literacy. J Gen Intern Med..

[31] Glasheen WP, Cordier T, Gumpina R (2019). Charlson Comorbidity Index: *ICD-9* Update and *ICD-10* Translation. Am Health Drug Benefits..

[32] **Schafer Jl, Graham JW.** Missing Data: Our view of the state of the art. *Psychological Methods.* 2002;7(2):147–177. 12090408

[33] **Schumacker RE, Lomax RG.** A Beginner’s Guide to Structural Equation Modeling: Fourth Edition. Psychology Press. 2004. 10.4324/9781410610904

[34] **Awang Z.** Structural Equation Modeling Using Amos Graphic: UiTM Press. 2012.

[35] **Kline RB.** Principles and practice of structural equation modeling. Guilford Publications. 2015.

[36] Petersen MR, Deddens JA (2008). A comparison of two methods for estimating prevalence ratios. BMC Med Res Methodol..

[37] **Housten AJ, Hoover DS, Correa-Fernandez V, Strong LL, Heppner WL, Vinci C, et al.** Associations of Acculturation with English- and Spanish-Langauge Health Literacy Among Bilingual Latino Adults. *Health Literacy Research & Practice*. 2019;3(2). 10.3928/24748307-20190219-01 PMC6607768 31294309

[38] Sentell T, Braun KL (2012). Low Health Literacy, Limited English Proficiency, and Health Status in Asians, Latinos, and Other Racial/Ethnic Groups in California. Journal of Health Communication..

[39] **Pew Research Center.** Unemployment rate is higher than officially recorded, more so for women and certain other groups. https://www.pewresearch.org/fact-tank/2020/06/30/unemployment-rate-is-higher-than-officially-recorded-more-so-for-women-and-certain-other-groups/. Published 2020. Accessed November 10, 2022.

[40] Kimbro RT, Bzostek S, Goldman N, Rodriguez G (2008). Race, Ethnicity, And The Education Gradient in Health. Health Affairs..

[41] Kaushal N, Kaestner R (2005). Welfare Reform and Health Insurance of Immigrants. Health Serv Res..

[42] Barksdale CL, Rodick WH, Hopson R (2017). Literature of the National CLAS Standards: Policy and Practical Implications in Reducing Health Disparities. J Racial and Ethnic Health Disparities..

[43] **Department of Health and Human Services.** National action plan to improve health literacy [internet]. Washington DC: HHS, 2010 [cited 2022 Oct 5]. Available from: http://www.health.gov/communication/hlactionplan/pdf/Health_Literacy_Action_Plan.pdf

[44] Chen AH, Youdelman MK, Brooks J (2007). The legal framework for language access in health-care settings: Title VI and beyond. Journal of General Internal Medicine..

[45] **Gadon M.** Trained Interpreters: A necessary expense [Internet]. American Medical News; 2008 [cited 2022 Oct 5]. Available from: http://www.ama-assn.org/amednews/2007/12/03/prcal203.htm

[46] Rueda-Medina B, Gomez-Urquiza JL, Tapia-Haro R (2020). Assessing health science students’ health literacy and its association with health behaviors. Health Soc Care Community..

[47] **Derose KP, Bahney BW, Lurie N, et al.** Review: Immigrants and Health Care Access, Quality, and Cost. *Medical Care Research and Review*. 2009;66(4). 10.1177/1077558708330425 19179539

[48] Leslie CJ, Donelan K, Nicholas P (2022). Health Literacy Profiles of Early Intervention Providers: Use of the Health Literacy Questionnaire. Health Lit Res Prac..

[49] **Naperola-Johnson J, Gutierrez J, Doyle K, et al.** Implementation of health literacy training for clinicians in a federally qualified health center. *PEC Innovation*. 2022;100083. 10.1016/j.pecinn.2022.100083 PMC10194109 37213779

[50] Allenbaugh J, Spagnoletti CL, Rack L (2019). Health literacy and clear bedside communication: A curricular intervention for internal medicine physicians and medicine nurses. MedEdPORTAL..

[51] Noordman J, Roodbeen R, Gach L, Schulze L, Rademakers J, van den Muijsenberg M (2022). ‘A basic understanding’; evaluation of a blended training programme for healthcare providers in hospital-based palliative care to improve communication with patients with limited health literacy. BMC Med Educ..

[52] Gibson C, Smith D, Morrison AK (2022). Improving Health Literacy Knowledge, Behaviors, and Confidence with Interactive Training. Health Lit Res Pract..

[53] Fragomen AT (1997). The Illegal Immigration Reform and Immigrant Responsibility Act of 1996: An Overview. Int Migr Rev..

[54] **Tuition and Financial Aid Equity for Undocumented Students.** Updated January 1^st^, 2023. Accessed January 6^th^, 2023. Accessible from: ttps://www.higheredimmigrationportal.org/states/

[55] Pope NG (2016). The Effects of DACAmentation: The Impact of Deferred Action for Childhood Arrivals on Authorized Immigrants. Journal of Public Economics..

[56] Venkataramani AS, Shah SJ, O’Brien R, Kawachi I, Tsai AC (2017). Health Consequences of the US Deferred Action for Childhood Arrivals (DACA) Immigrant Programme: a quasi-experimental study. Lancet Public Health..

[57] **US Code Title VIII**, Chapter 14 – Restricting Welfare and Public Benefits for Aliens. 8 U.S.C. § 1621 (2012). https://www.govinfo.gov/app/details/USCODE-2011-title8/USCODE-2011-title8-chap14-subchapII-sec1621/context.

[58] **New York State Education Department**, Press Release: Board of Regents Permanently Adopts Regulations to Allow DACA Recipients to Apply for Teacher Certification and Professional Licenses. Updated May 17^th^, 2016. Accessed January 6^th^, 2023. Accessible from: http://www.nysed.gov/news/2016/board-regents-permanently-adopts-regulations-allow-daca-recipients-apply-teacher.

[59] Sorenson K, Van den Broucke S, Fullam J, Doyle G, Pelikan J, Slonska Z (2012). Health literacy and public health: A systematic review and integration of definitions and models. BMC Public Health..

[60] Paasche-Orlow MK, Wolf MS (2007). The causal pathways linking health literacy to health outcomes. Am J Health Behav..

[61] Baker DW (2006). The Meaning and Measure of Health Literacy. J Gen Intern Med..

