# Supplementary material for: Predicted Health Literacy Disparities Between Immigrant and US-Born Racial/Ethnic Minorities: a Nationwide Study
Source: J Gen Intern Med. 2023 Feb 27;38(10):2364–73. doi: 10.1007/s11606-023-08082-x (PMC10406741; doi:10.1007/s11606-023-08082-x)
Supplement: Supplementary file 1 — Supplementary file1 (DOCX 57 KB) [file 11606_2023_8082_MOESM1_ESM.docx]

**Supplementary Materials**

**Figure S1. Latent Variable Structure – Health System Resource and Use**

**
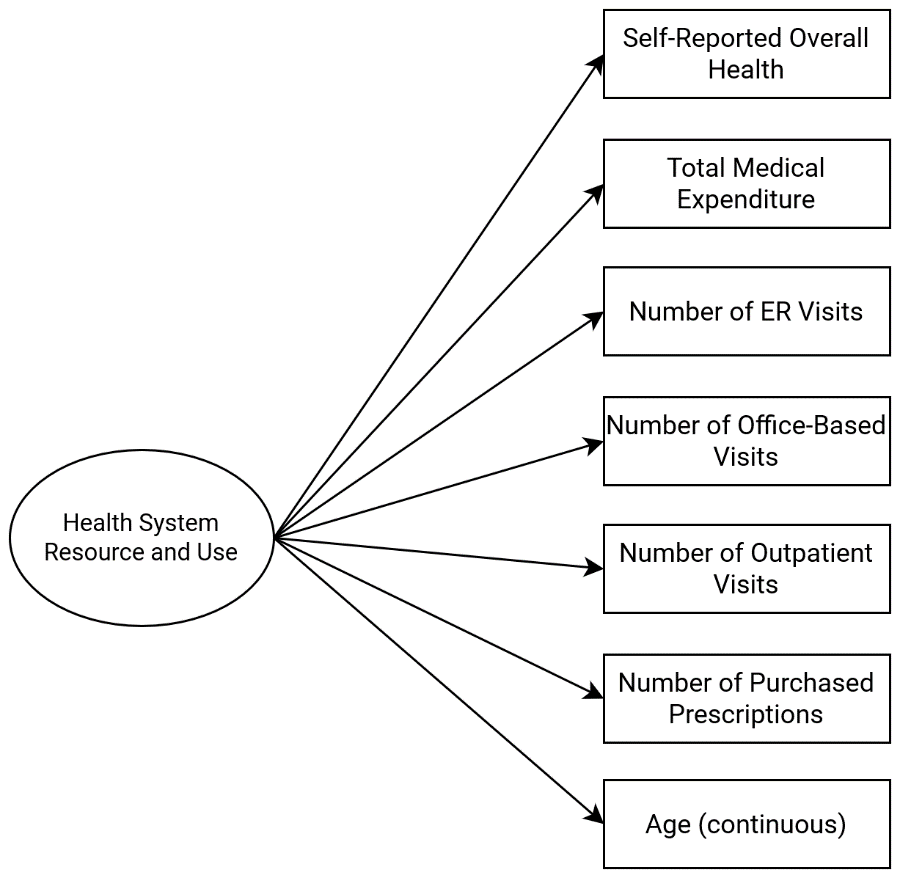
**

**Table S1. Path Analysis Results: Indirect Path Standardized Coefficients**

| Pathway | Standardized Coefficient | p-value |
| --- | --- | --- |
|  |  |  |
| **To Predicted HL Score** |  |  |
| Unemployed | 0.0020 | 0.184 |
| Insurance Coverage | 0.028 | <0.05 |
| Immigrant Birth Status | -0.018 | <0.05 |
| Education (HS or Higher) | 0.046 | <0.05 |
| No Cognitive Limitation | 0.079 | <0.05 |
| Visual Impairment | -0.018 | <0.05 |
| Hearing Impairment | -0.021 | <0.05 |
| Unmarried | 0.00081 | 0.435 |
| Metropolitan Residence | 0.0086 | <0.05 |
| **To Insurance Coverage** |  |  |
| Education (HS or Higher) | -0.020 | 0.019 |
| No Cognitive Limitation | -0.023 | 0.008 |
| **To Health System Use** |  |  |
| Unemployed | -0.0099 | 0.14 |
| Immigrant Birth Status | -0.016 | <0.05 |
| Education (HS or Higher) | -0.014 | 0.159 |
| No Cognitive Limitation | 0.00089 | 0.606 |
| Unmarried | -0.012 | <0.05 |

HS: High School

**Section S1: Questions Used to Assess Cognitive, Visual, and Auditory Limitations**

The following section describes the original survey questions used to collect MEPS participant data for cognitive limitations, visual challenges, or hearing challenges:

Cognitive Limitations:

The Agency for Healthcare Research, and Quality (AHRQ) collects data for MEPS using a combination of individual participant surveys, and surveys of a participant’s household (the members primarily living in one household unit surveyed). The variable indicating cognitive limitation was collected at this family level, asking if any “of the adults in the family experience confusion or memory loss, have problems making decisions, or require supervision for their own safety”. If a family unit answered “yes”, then the individual in question was identified and coded as “yes”. All other family members were coded as “no”.

Visual Challenges:

The survey question used to identify individuals with visual challenges was first asked at the family-unit level: “Does anyone in the family have difficulty seeing?”. This was followed by determination of the individual in question with a visual impairment. This individual was coded as “yes”, with the other family members coded as “no”.

Hearing Challenges:

The survey question used to identify individuals with hearing challenges was first asked at the family-unit level: “Does anyone in the family have difficulty hearing?”. This was followed by determination of the individual in question with a hearing impairment. This individual was coded as “yes”, with the other family members coded as “no”.
